# Microalgae and Cyanobacteria Species Valorization Towards Sustainable Bioplastics Production

**DOI:** 10.3390/md24100367

**Published:** 2026-10-20

**Authors:** Eirini Sventzouri, Savvas Giannis Mastropetros, Konstantinos Pispas, Sameh S. Ali, Michael Kornaros

**Affiliations:** 1 Laboratory of Biochemical Engineering & Environmental Technology (LBEET), Department of Chemical Engineering, University of Patras, 26504 Patras, Greece; eirinisventzouri@gmail.com (E.S.); savvasgiannismas@gmail.com (S.G.M.); pispas.msci@gmail.com (K.P.); 2 Botany Department, Faculty of Science, Tanta University, Tanta 31527, Egypt; samh_samir@science.tanta.edu.eg

**Keywords:** microalgae, cyanobacteria, bioplastics, polyhydroxyalkanoates, acetate, anaerobic digestion effluent, wastewater bioremediation, nitrogen starvation

## Abstract

The depletion of fossil resources and increasing plastic-derived pollution have become major environmental concerns. In this context, biodegradable biopolymers such as polyhydroxyalkanoates (PHA) have attracted considerable scientific interest. Microalgae and cyanobacteria represent promising photosynthetic microorganisms for sustainable bioplastic production. In this study, ten microalgal species (*Parachlorella kessleri*, *Chlorella vulgaris*, *Chlorella minutissima*, *Tetraselmis tetrathele*, *Chromochloris zofigiensis*, *Acutodesmus obliquus*, *Euglena gracilis*, *Chlorococcum oleofaciens*, *Botryococcus braunii*, and *Coelastrella vacuolata*) and four cyanobacterial species (*Arthrospira platensis*, *Nostoc muscorum*, *Chlorogloeopsis fritschii*, and *Synechocystis* sp.) were screened for growth and PHA accumulation. Cultivation was performed under autotrophic conditions, mixotrophic growth with acetate, mixotrophic growth in anaerobic digestion effluent, and nitrogen-depleted conditions. *Arthrospira platensis* showed the highest PHA content under autotrophic growth, reaching 4.3% *w*/*w*. Most species adapted successfully to anaerobic digestion effluent, achieving total nitrogen and phosphorus removal above 90% in most cases, although PHA accumulation remained low (0.6–1.3% *w*/*w*). Acetate supplementation and nitrogen limitation markedly enhanced PHA accumulation. Mixotrophic cultivation with acetate yielded up to 10.2% *w*/*w* in cyanobacteria, whereas nitrogen starvation increased accumulation further, with *Synechocystis* sp. reaching 14.4% *w*/*w*. Overall, cyanobacteria outperformed green microalgae, supporting their potential for sustainable bioplastic production. These findings provide a basis for species selection and future process optimization.

## 1. Introduction

The widespread use of petrochemical-derived plastics has led to severe environmental challenges, which establish plastic pollution as a global crisis. Conventional plastics, synthesized from fossil resources, are highly resistant to degradation and accumulate in terrestrial and aquatic ecosystems [1]. Reports of plastic debris in marine environments go back about fifty years, with continuous buildup on the ocean surface observed over the past six decades. Plastic emissions are rising and are expected to keep increasing, even under some of the most optimistic scenarios for reducing plastic waste [2]. This persistence contributes to the formation of microplastics, which infiltrate food webs and pose risks to both environmental and human health. Human exposure to and ingestion of microplastics have been associated with a growing range of health disorders [3]. The potential scale of these effects is highlighted by a survey from 2014, which estimated that a person may consume up to 11,000 tiny plastic particles per year [4]. Additionally, the production and degradation of these materials is closely linked to greenhouse gas emissions and resource depletion, further exacerbating climate change [5]. The urgent need to transition toward sustainable materials has driven significant interest in biodegradable and bio-based alternatives.

Plastic production and consumption have grown exponentially in recent decades. For example, global plastic output reached 413.8 million metric tons in 2023, a dramatic increase compared to just 1.5 million metric tons in 1950 [6]. Bioplastics have emerged as promising substitutes for conventional plastics, offering reduced environmental impact and improved end-of-life options. Among them, polyhydroxyalkanoates (PHA) represent a versatile family of biodegradable polyesters synthesized by various microorganisms as intracellular carbon and energy storage compounds. Two of the most studied members are poly(3-hydroxybutyrate) (PHB) and poly(3-hydroxybutyrate-*co*-3-hydroxyvalerate) (PHBV or PHB-V), which differ in their monomer composition and resulting material properties. Compared to petrochemical plastics such as polyethylene or polypropylene, PHA are biodegradable, biocompatible, and can be produced from renewable resources. However, their large-scale commercialization is still constrained by high production costs and limited productivity [7].

Microalgae and cyanobacteria have gained increasing attention as sustainable platforms for PHA production due to their ability to convert carbon dioxide into valuable biopolymers through photosynthesis [8]. These photosynthetic microorganisms can accumulate PHA under specific environmental conditions, particularly when subjected to nutrient limitation (e.g., nitrogen or phosphorus deprivation) in the presence of excess carbon. The metabolic pathways involved in PHB synthesis are relatively well understood, typically involving the conversion of acetyl-CoA into PHB via key enzymes such as 3-ketothiolase, acetoacetyl-CoA reductase, and PHB synthase. In cyanobacteria, carbon fixation through the Calvin cycle provides the precursors for PHA biosynthesis, linking polymer production directly to photosynthetic activity. Despite these advantages, challenges remain in optimizing yields and scaling up cultivation systems [9].

Around 100 microalgae and cyanobacteria species have been evaluated for PHA accumulation, with PHB content ranging between 0.04% and 6% of their dry weight [10]. While autotrophic PHA production from cyanobacteria and a few microalgae has been reported, their content in biomass remains relatively low [11]. Different strategies have been reported to enhance PHA accumulation. Supplementation of organic carbon sources such as acetate, valerate, glucose, and fructose has been reported to increase PHA content [12,13]. Nutrients limitation, such as nitrogen and phosphorus, also promotes PHA biosynthesis as cells redirect carbon excess to storage compounds [14]. Specifically, nitrogen starvation has been reported to increase PHB yield more than 50% in different cyanobacterial species [15]. In cyanobacteria, nitrogen limitation also induces glycogen accumulation as a carbon energy storage compound. During prolonged nitrogen starvation, glycogen is progressively mobilized to provide carbon precursors for PHB biosynthesis [16].

While PHA production from photosynthetic microorganisms is promising due to high growth rates, CO_2_ sequestration, and biorefinery potential, allowing simultaneous production of valuable compounds like lipids, proteins, polyunsaturated fatty acids, and pigments, its commercialization remains unfeasible [17,18]. Their production remains significantly more expensive than conventional plastics, due to the high costs of nutrient supplementation and organic carbon sources, which account for 45–50% of the total cost [19,20]. To address economic and sustainability limitations, the use of alternative, low-cost substrates has been widely investigated. Anaerobic effluents, commonly referred to as digestates, are nutrient-rich by-products of anaerobic digestion processes used in waste treatment and bioenergy production. These streams contain significant amounts of nitrogen, phosphorus, and organic carbon, making them attractive candidates as growth media for microalgae and cyanobacteria [21]. Utilizing digestate not only reduces the reliance on synthetic fertilizers but also contributes to nutrient recycling and wastewater remediation [22]. This integrated approach supports circular economy principles by coupling waste valorization with bioplastic production. In this context, the cultivation of microalgae and cyanobacteria in anaerobic effluents represents a promising strategy for sustainable PHA production. By simultaneously addressing waste management, nutrient recovery, and biopolymer synthesis, this approach can significantly lower production costs and environmental impacts [23].

Although PHA accumulation has been reported in several photosynthetic microorganisms, research has mainly focused on cyanobacteria, including genera such as *Synechocystis, Arthrospira*, *Synechococcus*, and *Nostoc*, while bioplastic production of many eukaryotic microalgal species remains comparatively unexplored [24,25]. Furthermore, although the cultivation of photosynthetic microorganisms in anaerobic effluents for waste bioremediation has been extensively investigated [26], the cultivation of microalgae and cyanobacteria in digestates for PHA production is rather scarce [27]. Therefore, cultivation of different microalgae and cyanobacteria species under different conditions is essential to evaluate how strain-specific characteristics and cultivation conditions affect biopolymer production.

The aim of this study is the evaluation of ten microalgal and four cyanobacterial species in terms of their ability to accumulate PHA under different cultivation conditions. A comprehensive screening was conducted under (i) autotrophic conditions, (ii) mixotrophic conditions with acetate, (iii) mixotrophic conditions with 10% *v*/*v* digestate, and (iv) nitrogen-free synthetic medium. This comparative approach was designed to identify the most promising strains in terms of growth and intracellular PHA accumulation, and to gain insights into the metabolic mechanisms underlying PHA biosynthesis under distinct environmental conditions. The results of this screening provide a basis for selecting the most suitable microalgal or cyanobacterial species for further optimization and bioplastic production. The photosynthetic microorganisms that participated in the screening process were: *Botryococcus braunii*, *Chlorella vulgaris*, *Chlorella minutissima*, *Parachlorella kessleri*, *Acutodesmus obliquus*, *Chromochloris zofigiensis*, *Chlorococcum oleofaciens*, *Euglena gracilis*, *Tetraselmis tetrathele*, *Coelastrella vacuolata*, *Chlorogloeopsis fritschii*, *Arthrospira platensis*, *Nostoc muscorum*, and *Synechocystis* sp. Many of these species, such as *C. minutissima*, *T. tetrathele*, *C. vulgaris*, *P. kessleri*, *C. vacuolata*, *A. obliquus*, and *A. platensis*, can also be cultivated in seawater [28]. The ability of these species to adapt to saline environments broadens their potential for sustainable bioplastic production while reducing dependence on freshwater resources.

## 2. Results and Discussion

### 2.1. Autotrophic Screening

**Figure 1:**
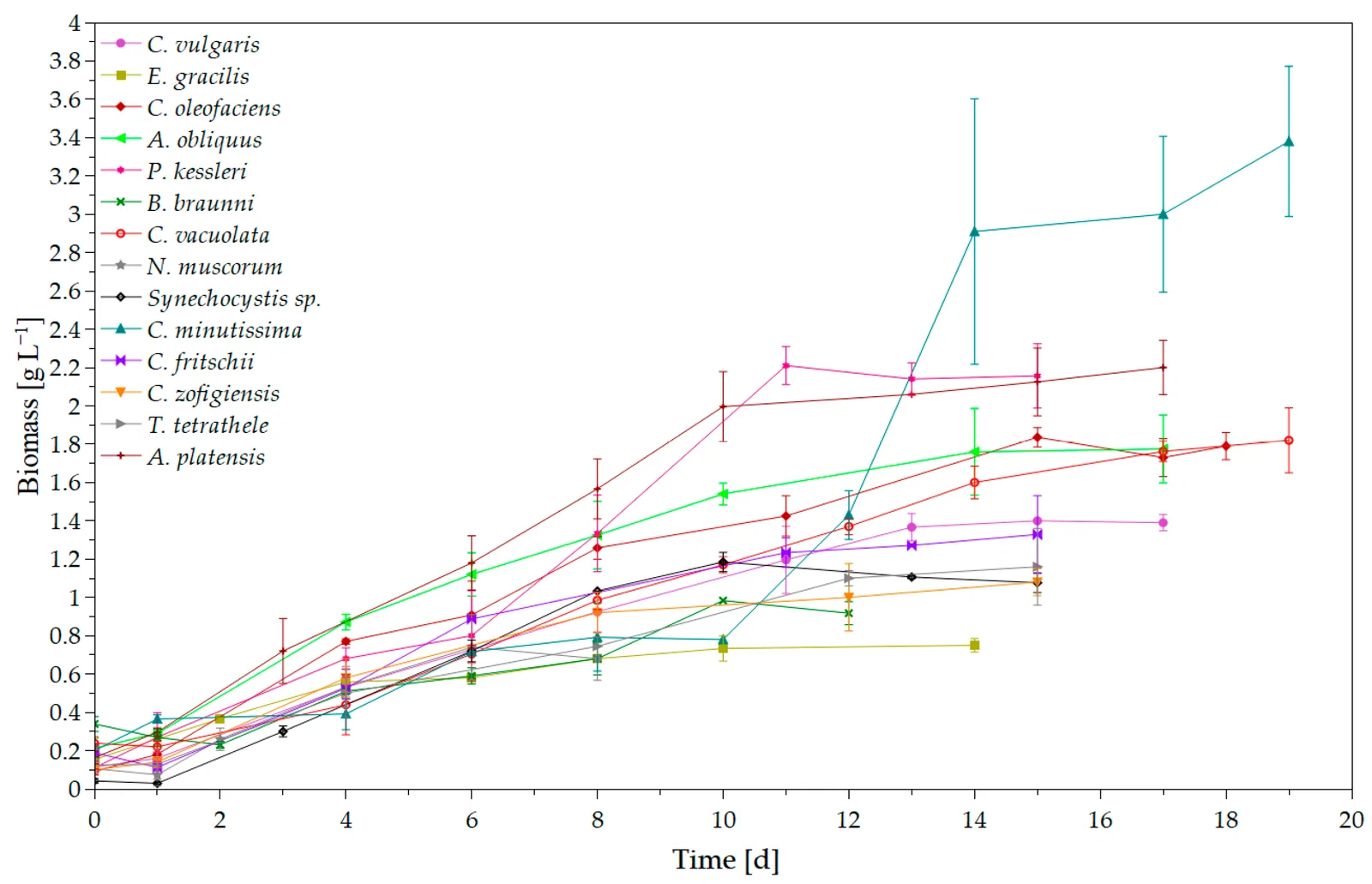
Biomass concentration of microalgal and cyanobacterial species during autotrophic cultivation.

Figure 1 presents the biomass concentration of photosynthetic microorganisms during autotrophic cultivation. The highest biomass concentration was observed for the microalgae *C. minutissima* (3.4 g L^–1^), while *P. kessleri* and *A. platensis* reached around 2.0 g L^–1^ by day 10 of cultivation. High biomass concentrations, ranging between 1.7 and 3.1 g L^–1^ at different pH values, have also been reported for *A. platensis* [29]. In the study of Chakraborty et al. [30], biomass concentration of *C. minutissima* was equal to 1.2 g L^−1^, during autotrophic cultivation under similar cultivation conditions considering temperature and light intensity. The lowest biomass concentration was observed for *E. gracilis* (0.8 g L^−1^), *B. braunii* (0.9 g L^−1^), and *N. muscorum* (0.7 g L^−1^). Nutrient removal varied among the species (Table 1). For species cultivated in BG-11 medium initial concentrations of total nitrogen (TN) and total phosphorus (TP) were 250 and 25 mg L^−1^, respectively. For *N. muscorum, A. platensis*, and *E. gracilis* initial TN concentrations were 28, 412, and 28 mg L^−1^, while initial TP concentrations were 4, 89, and 5 mg L^−1^, respectively. TN removal ranged from 26.7% for *C. vacuolata* to 65.2% for *E. gracilis*, whereas TP removal exceeded 90% for most species. The relatively higher TN removal observed for cyanobacterial species may be associated with their nitrogen-rich biomass. Cyanobacteria, such as *Synechocystis*, can accumulate high protein levels, while nitrogen uptake is also used for phycobiliprotein synthesis [31,32]. In cultures that nutrients were still available, growth limitation was probably caused due to high cell density. As biomass concentration increases, light availability decreases resulting in a lower growth rate due to self-shading within the culture [33].

**Figure 2:**
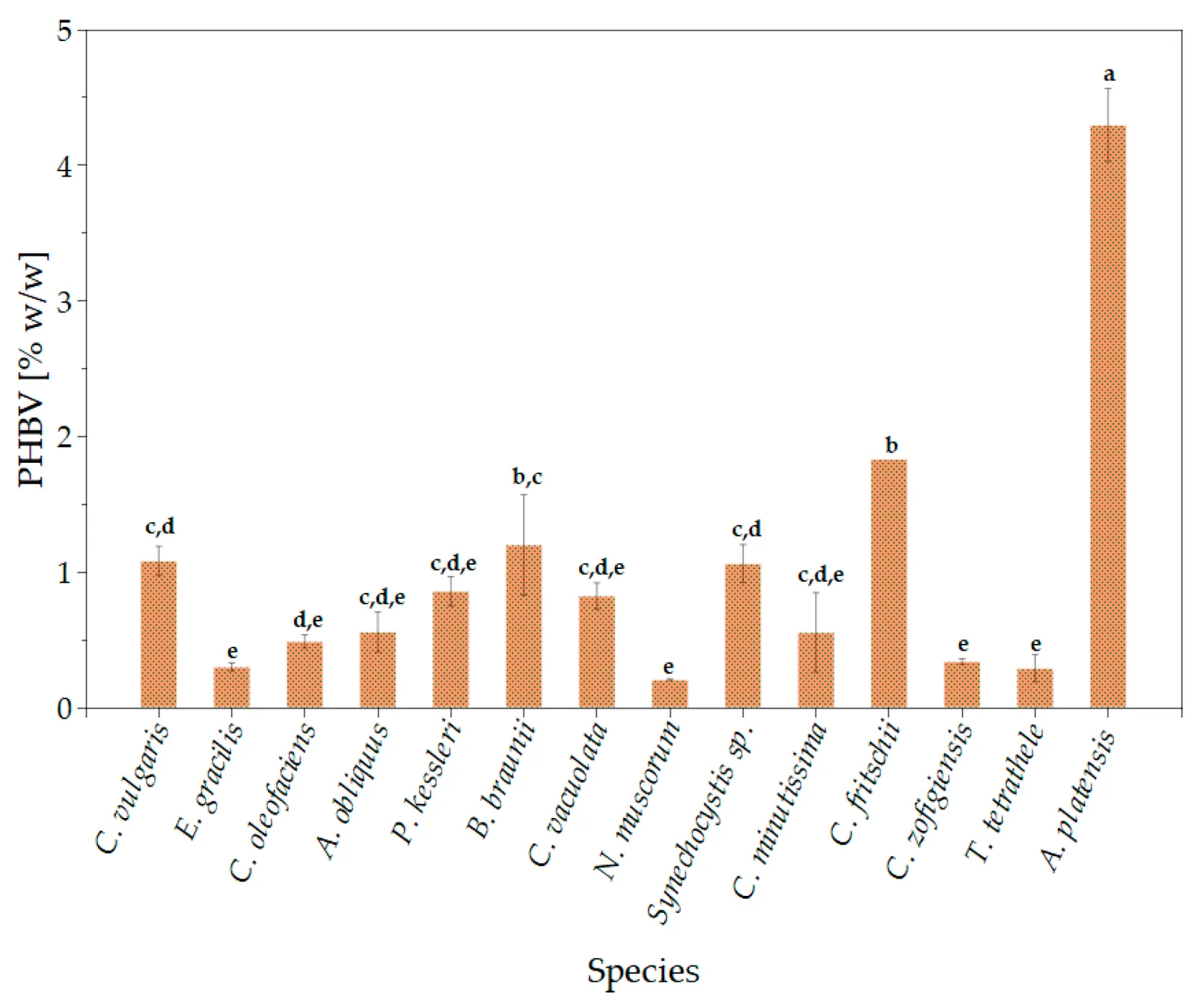
PHBV content (% *w*/*w*), on dry basis, of microalgal and cyanobacterial species during autotrophic cultivation. ^a,b,c,d,e^ Data that do not share a letter are significantly different based on Tukey test (*p* < 0.05).

At the end of cultivation, PHBV accumulation was determined (Figure 2). A. *platensis* accumulated the highest bioplastic content equal to 4.3% *w*/*w*, with a 3HV fraction of 14.3 mol%, followed by *C. fritschii* (1.8% *w*/*w*) and *B. braunii* (1.2% *w*/*w*). In the study of Zhila et al. [12], *S. platensis* accumulated 0.9% *w*/*w* PHA during the stationary phase of cultivation in a standard medium. Cassuriaga et al. [34] reported that PHB production in *Chlorella fusca* LEB 111 was undetectable after 15 days of cultivation in both standard BG-11 medium and nitrogen-reduced treatments. Similar results were reported by Kamravamanesh et al. [35], where *Synechocystis* sp. PCC 6714 grown in complete BG-11 accumulated PHB content equal to 1% *w*/*w*. In general, autotrophic PHA production is generally constrained by low yields [12]. Higher PHA production during autotrophic growth has been reported for locally isolated species. *B. braunii* (isolated from the fresh water of Kolavoi lake in Chengalpattu) accumulated almost 20% *w*/*w* in CHU-13 medium [36]. Similarly, *Spirulina* LEB 18 (isolated from Mangueira Lagoon located in Santa Vitoria do Palmar/RS) reached a crude biopolymer efficiency of 21% *w*/*w* during 15 days of cultivation in Zarrouk medium [37]. These differences in PHA accumulation can be attributed to the metabolic variations among different strains, as well as the cultivation conditions. Both strains were cultivated under 12/12 light/dark photoperiod, while *Spirulina* was also cultivated at 30 °C and at lower light intensity (41.6 μmol m^−2^ s^−1^). Light intensity and photoperiod affect bioplastic accumulation depending on the strain. In the study of Fleischhacker-Daffert et al. [38], continuous illumination at moderate light intensity led to an increased PHB accumulation in *Synechocystis salina* CCALA 192 compared to day/night cycles.

### 2.2. Mixotrophic Screening with Acetate

**Figure 3:**
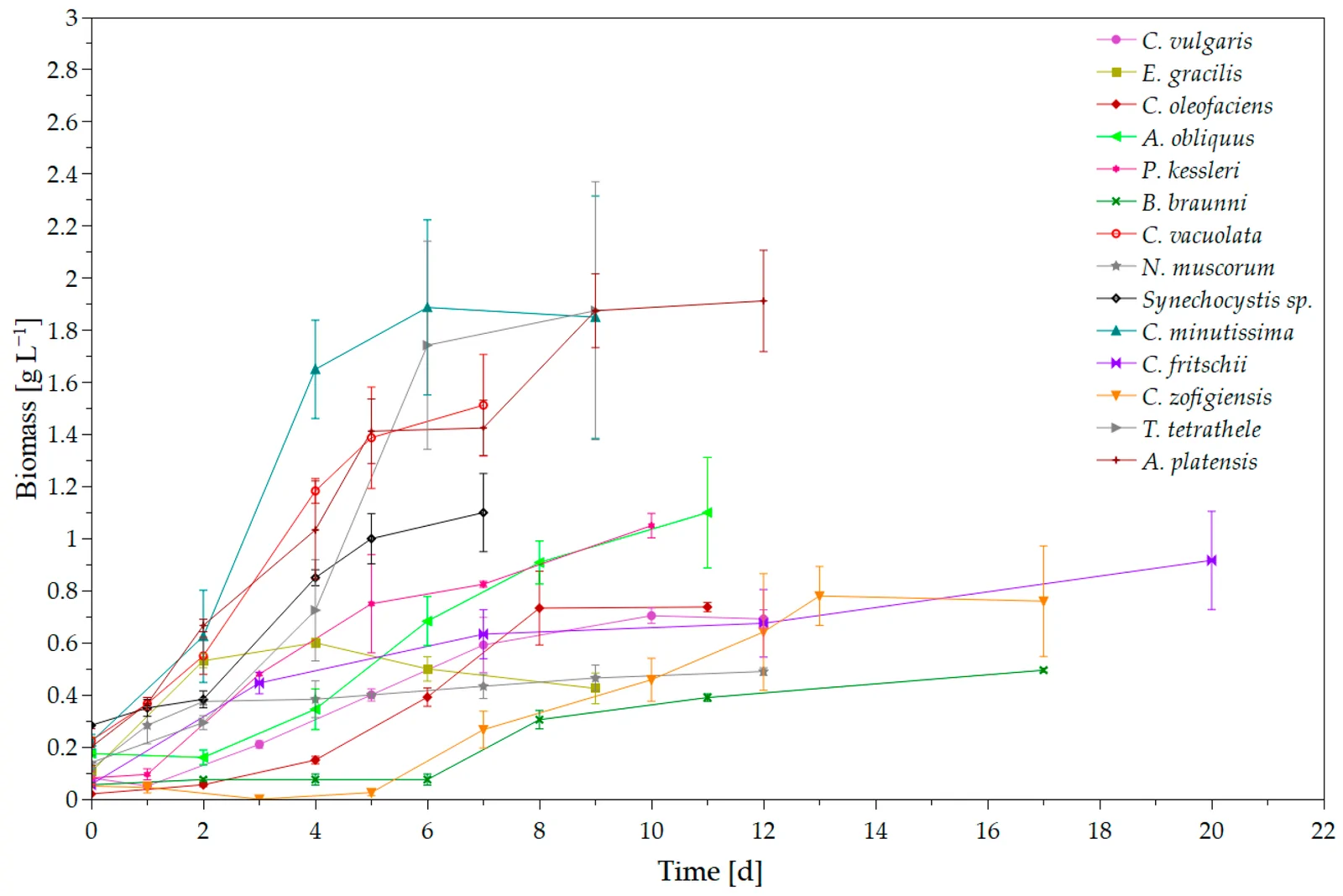
Biomass concentration of microalgal and cyanobacterial species during mixotrophic cultivation with acetate.

Biomass concentration during mixotrophic growth with acetate is presented in Figure 3. *C. minutissima* exhibited the most rapid biomass growth, reaching almost 1.9 g L^−1^ by day 6. *A. platensis* and *C. vacuolata* also began to grow quickly, achieving maximum biomass concentrations of 1.9 and 1.7 g L^−1^, respectively. Several studies have demonstrated that acetate supplementation enhances the growth of *A. platensis* [39]. Matsudo et al. [40], reported a 39% increase in the biomass concentration of *A. platensis*, reaching 1.8 g L^−1^, when using acetate in fed-batch cultivation compared to autotrophic growth. Acetate has also been shown to enhance biomass productivity of *C. minutissima*, although excessive acetate concentrations may become inhibitory, indicating that the response depends strongly on substrate concentration and strain physiology [41]. In general, mixotrophic cultivation allows cells to simultaneously use light and CO_2_ through photosynthesis and an external organic carbon source through respiration and heterotrophic metabolism. The simultaneous operation of these metabolic pathways can increase the availability of carbon and energy for biomass formation, potentially resulting in higher biomass concentration [42]. In contrast, *C. oleofaciens*, *C. zofigiensis*, and *B. braunii* displayed a lag phase of approximately 4–6 days before growth and biomass concentrations ranging between 0.5 and 0.7 g L^−1^. This delayed adaptation following organic carbon exposure likely reflects the period required for metabolic acclimation, as well as the regulation of acetate transport and utilization pathways. Abiusi et al. [43], reported a 48-h lag phase when autotrophically grown *Chlorella sorokiniana* was inoculated into a medium containing acetate, while acetate concentration and culture pH have also been shown to influence the duration of the lag phase [44].

Acetate removal demonstrated the different responses of the photosynthetic microorganisms to the addition of organic carbon (Table 2). Complete removal was achieved for *A. obliquus*, *N. muscorum*, and *Synechocystis* sp., while *P. kessleri* managed to remove only 9.1% of the acetate, probably due to its preference for autotrophic growth. However, acetate utilization was not directly reflected in cell division, indicating that could also be directed towards maintenance or intracellular storage. For instance, acetate supplementation has been reported to enhance lipid accumulation in *C. vulgaris*, with the response strongly depending on acetate concentration [45]. Compared to autotrophic growth, in most species TN removal increased, while TP removal decreased. A similar dependence on nutrient uptake has previously been reported for *C. vulgaris*, where ammonium uptake was enhanced under organic carbon supplementation, while phosphate uptake was higher under autotrophic conditions. This was attributed to the increased availability of adenosine triphosphate (ATP) and reduced nicotinamide adenine dinucleotide phosphate (NADPH), derived from organic carbon metabolism, which provided the energy and reducing power required for nitrogen assimilation under mixotrophic conditions. In contrast, the higher autotrophic phosphorus removal was associated with polyphosphate accumulation and efficient ATP synthesis [46].

**Figure 4:**
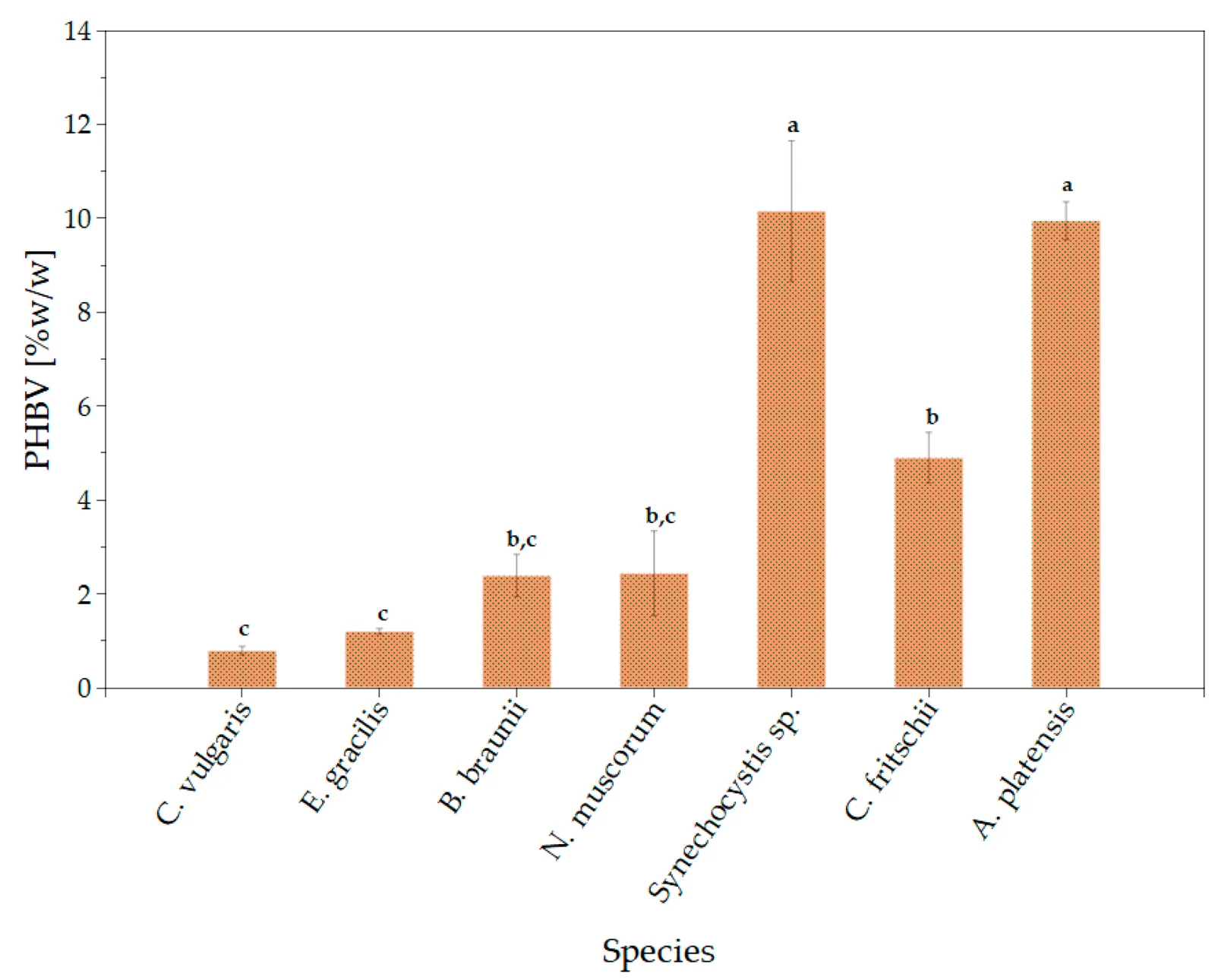
PHBV content (% *w*/*w*), on dry basis, of microalgal and cyanobacterial species during mixotrophic cultivation with acetate. ^a,b,c^ Data that do not share a letter are significantly different based on Tukey test (*p* < 0.05).

Acetate supplementation also affected bioplastic accumulation (Figure 4). PHBV content significantly increased in cyanobacteria *Synechocystis* sp., *A. platensis*, and *C. fritschii* reaching 10.2%, 9.9%, and 4.9% *w*/*w*, respectively. The corresponding 3HV fractions were 21.0 mol% in *Synechocystis* sp., 1.9 mol% in *A. platensis*, and 3.0 mol% in *C. fritschii*. In the study of Nematollahi et al. [23], the addition of 10 mM acetate increased PHB accumulation in *A. platensis* to approximately 3% *w*/*w* by day 8 of cultivation under a 12/12 light/dark photoperiod with a light intensity of 200 μmol m^−2^ s^−1^. In *C. fritschii*, acetate supplementation has previously increased PHB accumulation from approximately 5 to 15% of dry biomass [47]. Sharma and Mallick [48], reported that *N. muscorum* cultivated for 21 days in BG-11 medium supplemented with 0.2% *w/v* acetate, at 25 ± 1 °C, pH 8.0, under a 14:10 h light–dark cycle and an irradiance of 75 μmol photons m^−2^ s^−1^, accumulated 28% *w*/*w* PHB, compared with 8.6% *w*/*w* under photoautotrophic conditions. In general, the presence of acetate is expected to lead to increased bioplastic accumulation as it increases the intracellular levels of acetyl-CoA, which is the precursor for PHA biosynthesis [49]. Moreover, acetate supplementation increases the intracellular acetyl-CoA pool, while PHA synthesis can act as an electron sink, helping to rebalance the cellular ATP/NADPH ratio and conserve assimilated carbon [13]. However, PHBV remained low or was not detected in several other species despite measurable growth and, in some cases, extensive acetate removal. Therefore, the utilization of organic carbon alone was not sufficient to induce PHBV accumulation, emphasizing the species-specific partitioning of assimilated carbon between biomass formation and intracellular storage compounds.

### 2.3. Mixotrophic Screening in Anaerobic Digestion Effluent

**Figure 5:**
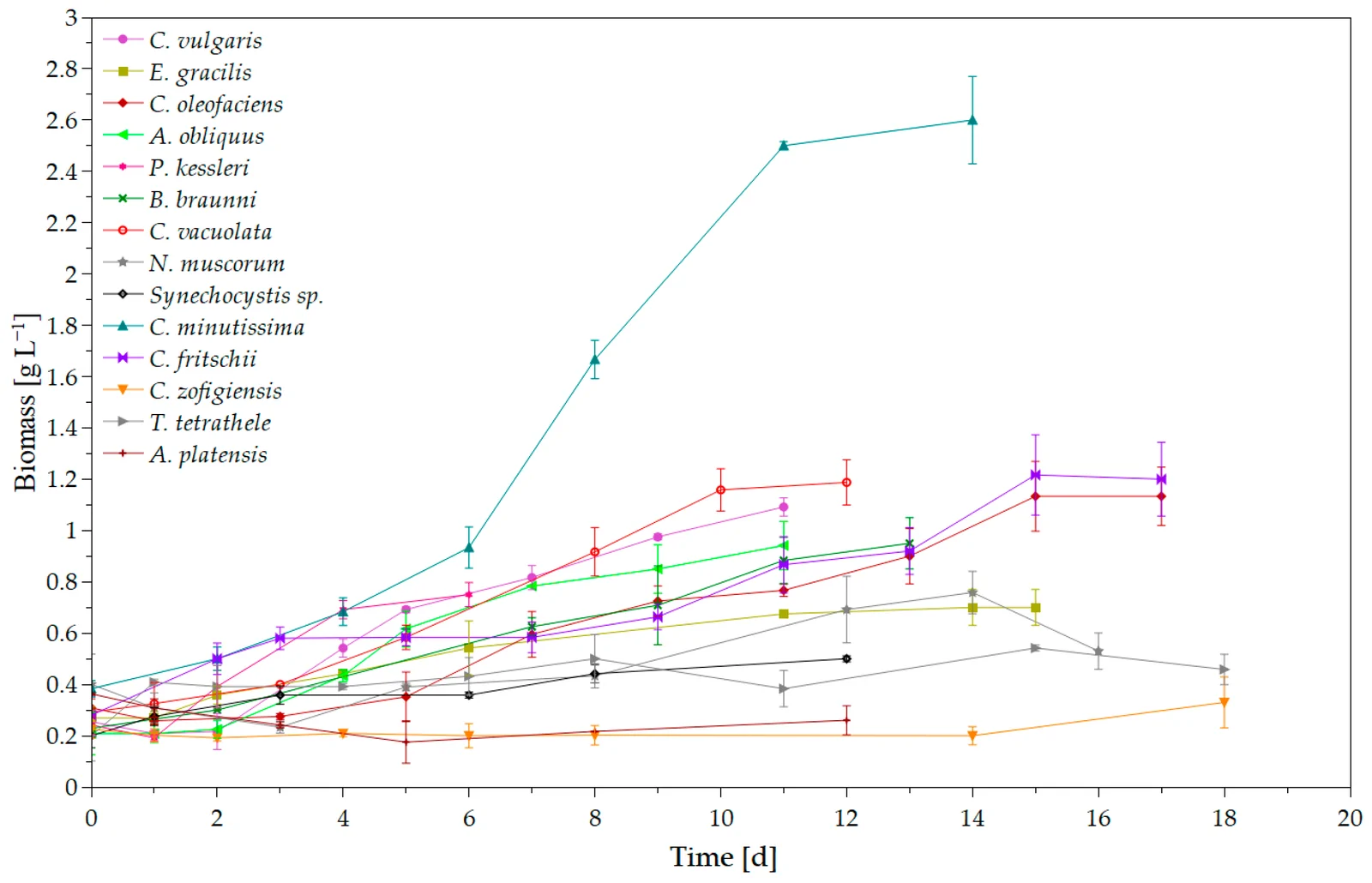
Biomass concentration of microalgal and cyanobacterial species during mixotrophic cultivation in 10% *v*/*v* anaerobic digestion effluent.

Mixotrophic screening was also performed in 10% *v*/*v* digestate. As shown in Figure 5, eleven out of fourteen species managed to adapt and grow in the wastewater stream, while a lag phase was observed for almost all species. However, *A. platensis*, *T. tetrathele*, and *C. zofigiensis* failed to grow. The highest biomass concentration was reported for the microalgae *C. minutissima*, equal to 2.6 g L^−1^. In the study of Singh et al. [50], the biomass of *C. minutissima* cultivated in 8% *v*/*v* poultry litter anaerobic digester effluent was 0.4 g L^–1^. Lower biomass concentrations were observed compared to the other cultivation methods, remaining below 1.2 g L^–1^, with the lowest values reported for the cyanobacterial species *Synechocystis* sp. and *N. muscorum* (0.5 g L^–1^). Similar results were reported in the study of Nur et al. [51], where biomass concentration of *N. muscorum* and *Synechocystis* sp. during cultivation in palm oil mill effluent was 0.5 and 0.6 g L^–1^, respectively. In another study [52], cultivation of *Synechocystis* sp. in 3% *v*/*v* digested municipal wastewater resulted in a biomass concentration of 1.5 g L^–1^, whereas increasing the effluent load to 12% decreased biomass concentration to almost 0.8 g L^–1^. The observed variations reflect the complex nature of digestate. While it supplies essential nutrients and organic carbon, it can also inhibit growth due to high ammonium levels, turbidity, reduced light penetration, and the presence of toxic compounds [53].

Digestate bioremediation was evaluated in terms of chemical oxygen demand (COD), TN, and TP removal (Table 3). TN removal exceeded 90% for most cultures, while TP removal reached 100% for *C. oleofaciens*, *A. obliquus*, *P. kessleri*, and *C. minutissima*. The TN removal efficiencies obtained in the present study were generally higher than those reported by Psachoulia et al. [54], who observed approximately 35–45% TN removal by different microalgal species cultivated in 3.7% *v*/*v* digestate originated from a biogas plant that processes animal husbandries despite the substantially lower initial TN concentration used in their study (approximately 170 mg L^−1^ compared with 378 mg L^−1^ in the present work). Ammonium is generally considered a readily assimilable nitrogen source for microalgae, with previous studies reporting inhibitory effects even within the range of 100–220 mg L^−1^ [55]. In the present study, despite an initial ammonium concentration of approximately 200 mg L^−1^, most species were able to grow, indicating a high tolerance to the applied cultivation conditions. However, not all the nitrogen may have been removed by the photosynthetic microorganism, since under alkaline conditions, ammonium nitrogen can be removed by ammonia stripping [26]. Many studies have reported high TP removal during the cultivation of various photosynthetic microorganisms in different types of liquid digestate [56,57,58,59]. However, the initial TP concentrations are often relatively low. The highest COD removal was reported for the cyanobacteria *N. muscorum* and *Synechocystis* sp. equal to 61.6% and 59.6%, respectively. However, COD removal remained relatively low for many species compared to the other nutrients. This is probably because the remaining organic matter in digestate after anaerobic digestion is composed of complex compounds that are difficult to break down [26].

**Figure 6:**
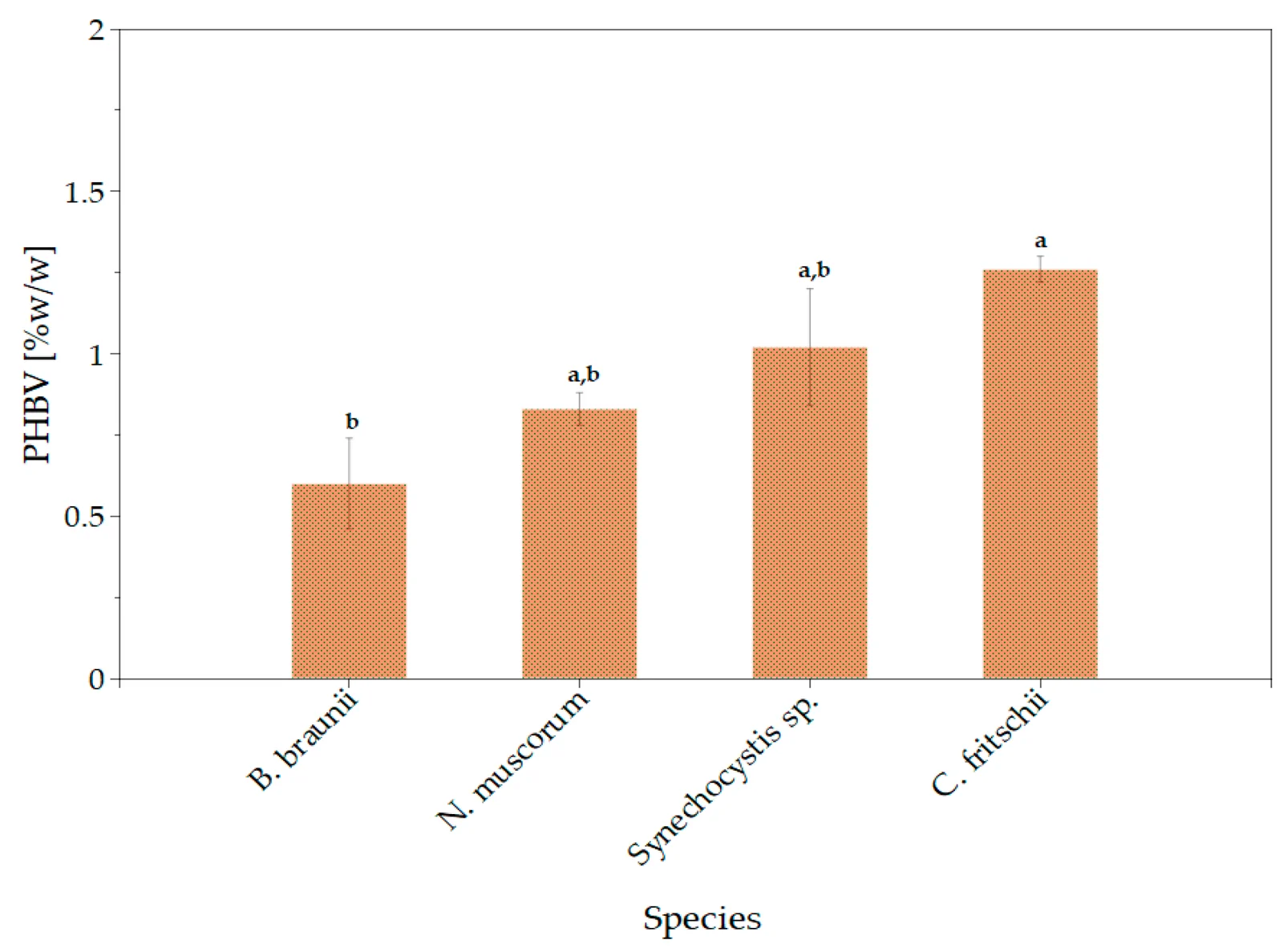
PHBV content (% *w*/*w*), on dry basis, of microalgal and cyanobacterial species during mixotrophic cultivation in 10% *v*/*v* anaerobic digestion effluent. ^a,b^ Data that do not share a letter are significantly different based on Tukey test (*p* < 0.05).

Mixotrophic cultivation in digestate did not favor bioplastic accumulation (Figure 6). Only four species were found to contain PHBV, with concentrations ranging from 0.6% to 1.3% *w*/*w*. Studies specifically addressing PHA production by photosynthetic microorganisms cultivated in anaerobic digestate remain limited. Meixner et al. [60], reported approximately 5.5% *w*/*w* PHB in *Synechocystis salina* CCALA 192 cultivated for 40 days in a 200-L tubular photobioreactor using 1:3 diluted supernatant of thin-stillage digestate derived from bioethanol production. Overall, cultivation in 10% *v*/*v* anaerobic digestion effluent was more effective for biomass production and wastewater treatment.

### 2.4. Screening in Nitrogen-Free Synthetic Medium

**Figure 7:**
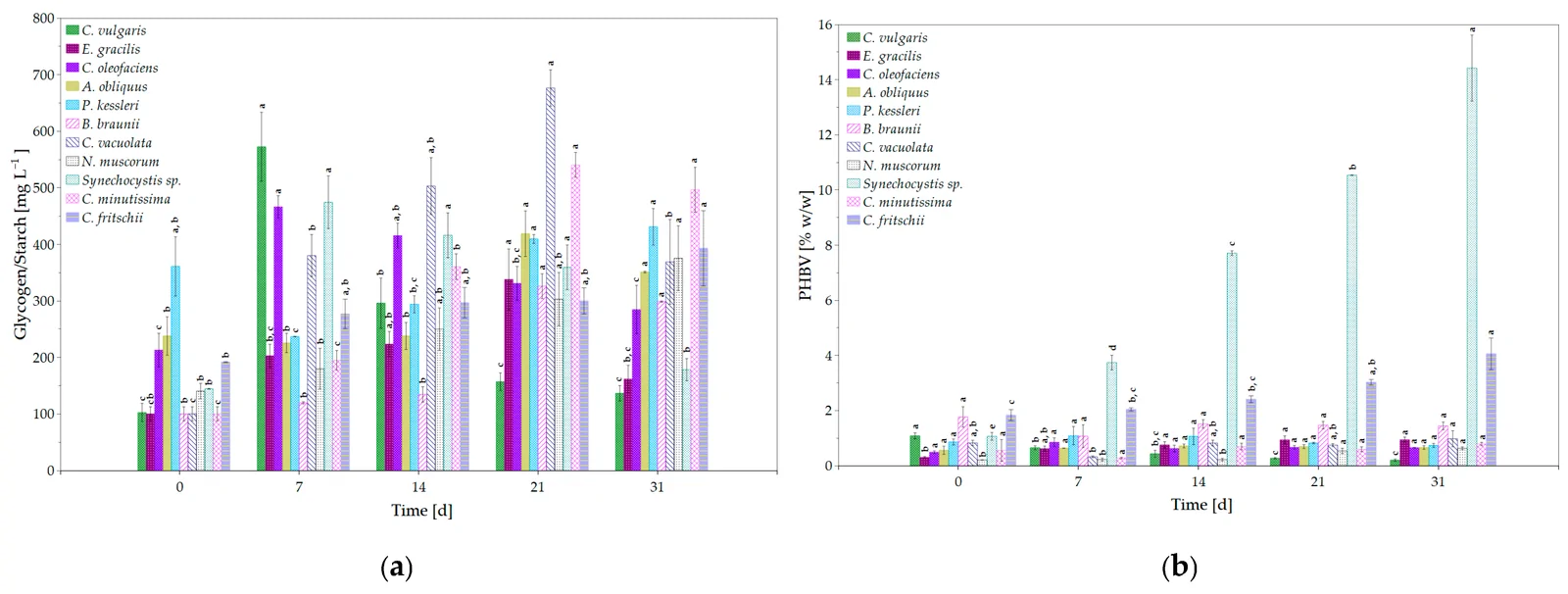
(**a**) Glycogen/Starch accumulation (mg L^−1^) and (**b**) PHBV content (%*w*/*w*) of microalgal and cyanobacterial species during cultivation in nitrogen-free synthetic medium. ^a,b,c,d,e^ Bars of a specific species at different days that do not share a letter are significantly different based on Tukey test (*p* < 0.05).

The species that successfully grew in digestate were further evaluated for their ability to accumulate bioplastic in nitrogen-free synthetic medium. Nitrogen limitation is widely reported to promote PHA biosynthesis by redirecting cellular carbon towards storage compounds [24,35,61]. In cyanobacteria, PHA biosynthesis is closely associated with central carbon metabolism and particularly with glycogen turnover, whereas microalgae primarily store carbohydrates as starch [16,62]. As shown in Figure 7, for *A. obliquus*, *P. kessleri*, *B. braunii*, and *C. minutissima*, starch content did not decrease during cultivation and PHBV content remained relatively stable. Similarly, *C. vulgaris*, *C. vacuolata*, *E. gracilis*, and *C. oleofaciens* showed substantial accumulation without a clear increase in PHBV. This indicates that, for the tested microalgae, starch accumulation was not directly associated with PHA biosynthesis. Among them, *B. braunii* showed the highest final PHBV content, reaching 1.4% *w*/*w*. In the study of Costa et al. [63], *C. minutissima* did not accumulate PHA under nitrogen depletion. PHB accumulation, reaching 30% *w*/*w*, has been reported for microalgal species such as *Scenedesmus* sp. and *Chlorella sorokiniana* in nitrogen-free synthetic medium but with the addition of an external organic carbon source [64,65]. A different response was observed for *Synechocystis* sp., which reached the highest PHBV content, equal to 14.4% *w*/*w*, on the last day of cultivation. The 3HV fraction decreased over the cultivation period, from 15.8 mol% at the beginning of nitrogen limitation to 1.8 mol% on the last day of cultivation. Such changes in PHBV monomer composition may reflect changes in carbon partitioning during the progression of nutrient limitation, as PHA composition can be affected by cultivation conditions and cellular metabolic state [66]. Glycogen increased significantly during the first days of cultivation and subsequently decreased, while PHBV continued to increase until day 31. This trend is consistent with the previously demonstrated role of glycogen turnover in PHB biosynthesis in *Synechocystis* sp. [16]. In other studies, *Synechocystis* sp. accumulated 4.1% and 9.5% *w*/*w* PHB after 7 and 21 days of nitrogen starvation, respectively [67,68]. In the case of *C. fritschii*, the glycogen content increased significantly on the last day of cultivation, with a bioplastic content of 4% *w*/*w*. A similar trend was observed for the 3HV fraction, which decreased from 15.7 mol% at the beginning of nitrogen limitation to 2.2 mol% on the last day of cultivation. However, during this period, no reduction in glycogen was observed. The cyanobacterium *N. muscorum* accumulated PHBV less than 1% *w*/*w*, while glycogen concentration did not decrease during the cultivation period. Although different strains of *N. muscorum* have been reported to accumulate PHA [48,69], *N. muscorum* is a nitrogen-fixing cyanobacterium which can make the establishment of nitrogen-limiting conditions challenging [70].

### 2.5. Comparative Analysis

As already observed, the different cultivation conditions affected each microorganism differently, both in terms of growth performance and accumulation of the target product. Table 4 presents the maximum growth rate, the maximum biomass concentration, and the bioplastic accumulated for each photosynthetic microorganism during the different screening experiments. Acetate generally promoted faster growth in several species, whereas *C. minutissima* showed the highest biomass concentration, reaching maximum values of 3.4, 1.9, and 2.6 g L^−1^ under autotrophic, acetate, and digestate cultivation, respectively. A two-way ANOVA revealed that the cultivation condition was the main source of variation in specific growth rate μ (45.42%), while maximum biomass was mainly determined by species identity (49.45%). The relatively high contribution of the species and cultivation condition interaction for both parameters further demonstrates that the response to each cultivation regime was strongly species dependent (Table A1). Moderate acetate supplementation can enhance growth. The maximum values of the specific growth rate and biomass concentration of *C. vulgaris* during mixotrophic cultivation with acetate were 0.75 d^−1^ and 3.5 g L^−1^, respectively, compared to 0.52 d^−1^ and 0.74 g L^−1^ observed during autotrophic growth [71]. However, high acetate concentrations can inhibit growth [72]. Although digestate can provide both nutrients and organic carbon, its complex composition may limit growth. Lower specific growth rates in anaerobic effluents than in synthetic media have also been reported [73,74]. Therefore, appropriate digestate pretreatment, including solid removal and optimization of the dilution ratio, is often required to mitigate inhibitory effects, improve light penetration, and create the appropriate conditions for growth.

In general, cyanobacteria outperformed green microalgae in terms of bioplastic accumulation. Among the eukaryotic photosynthetic microorganisms, *B. braunii* exhibited the highest PHBV content (2.4% *w*/*w*) when acetate was added. Cultivation conditions strongly affected PHBV accumulation in cyanobacteria. Compared to autotrophic cultivation, acetate supplementation increased PHBV approximately 9.3-fold in *Synechocystis* sp. (1.1% to 10.2% *w*/*w*), 2.7-fold in *C. fritschii* (1.8% to 4.9% *w*/*w*), and 2.3-fold in *A. platensis* (4.3% to 9.9% *w*/*w*). However, these comparisons should be interpreted with caution, given that the initial biomass concentration and light intensity differed under digestate and nitrogen-depleted conditions. Although higher light intensity was applied to account for medium characteristics and higher cell density, its potential influence on PHBV accumulation cannot be excluded. The highest PHBV content observed in the entire screening was produced by nitrogen depletion, with *Synechocystis* sp. reaching 14.4% *w*/*w*. Under nitrogen depletion, *C. fritschii* achieved a comparable content with acetate supplementation. In the study of Duangsri et al. [75], under nitrogen starvation, the PHBV accumulation in *A. platensis* reached 9.6% *w*/*w* and further increased to 19.6% *w*/*w* by supplementing 0.5% *w/v* acetate, significantly higher than the 1.9% *w*/*w* observed in the control. Similarly, in the study of Koch et al. [76], acetate supplementation increased PHB content 113%. In contrast, cultivation in digestate supported biomass production and substantial nutrient removal but did not result in a comparable enhancement of PHBV accumulation. Future optimization, through prolonged nutrient limitation or a two-stage cultivation approach, could enhance PHBV yields while maintaining the advantages of cultivation in wastewater. The 3HV fraction varied across the different cultivation conditions, with differences observed among both species and experimental treatments. Tarawat et al. [77], observed PHBV accumulation in several cyanobacterial strains cultivated with CO_2_ or acetate as the sole supplied carbon source, with 3HV fractions ranging from 4 to 24 mol% under acetate supplementation. One possible route involves the endogenous formation of propionyl-CoA through amino acid metabolism. Specifically, threonine can be converted to 2-oxobutyrate by threonine dehydratase, a reaction involved in isoleucine biosynthesis in *Synechocystis* sp. PCC 6803 [78]. Furthermore, the degradation of branched-chain amino acids, such as valine and isoleucine, has been associated with propionyl-CoA formation in green microalgae [79]. However, potential chromatographic interference or co-elution with other compounds present in the biomass cannot be excluded. Therefore, the detected 3HV fractions should be interpreted with caution, and further analytical verification is required to confirm their identity. The species-specific nature of PHBV accumulation was further confirmed by the statistical analysis, with species and the species × cultivation condition interaction explaining most of the observed variability (Table A1 and Table A2). Although cultivation conditions significantly affected PHBV accumulation (*p* < 0.001), their impact was strongly dependent on the photosynthetic microorganism.

## 3. Materials and Methods

### 3.1. Photosynthetic Microorganisms

The photosynthetic microorganisms examined in this study include several eukaryotic microalgae, *Parachlorella kessleri* SAG 211-11g, *Chlorella vulgaris* SAG 211-11b, *Chlorella minutissima*, *Tetraselmis tetrathele*, *Chromochloris zofigiensis* SAG 211-14, *Acutodesmus obliquus* SAG 276-6, *Euglena gracilis* SAG 1224-5/1, *Chlorococcum oleofaciens* SAG 213-11, *Botryococcus braunii* SAG 30.81, and *Coelastrella vacuolata* SAG 211-8b, as well as the cyanobacteria *Arthrospira platensis* SAG 21.99, *Nostoc muscorum* SAG 1453-12b, *Chlorogloeopsis fritschii* 1411-1a, and *Synechocystis* sp. PCC 6803. All strains were sourced from the SAG Culture Collection (University of Göttingen, Göttingen, Germany), except for *Synechocystis* sp., which was obtained from the Pasteur Culture Collection of Cyanobacteria (PCC), and *Chlorella minutissima*, which was isolated from the port of Heraklion, Crete, Greece [80].

The cells were maintained aseptically in 250 mL conical flasks containing synthetic media tailored to the specific requirements of each species. Most of the species were cultivated in BG-11 medium (73816, Sigma-Aldrich, St. Louis, MO, USA), as one of the most widely used synthetic media for the cultivation of various photosynthetic microorganisms [81], supplemented with a trace metal solution (Mix A5 with Co 92949, Sigma-Aldrich). Species with specific nutritional or salinity requirements were cultivated in their corresponding strain-specific media. Specifically, *B. braunii* required an additional 3% *v*/*v* soil extract, and *N. muscorum* was cultivated in Basal Medium (=ES “Erddekokt + Salze”), containing (g L^−1^) KNO_3_ (0.2), K_2_HPO_4_(0.02), MgSO_4_·7H_2_O (0.20), and 3% *v*/*v* soil extract, and 0.5% *v*/*v* micronutrient solution, containing the following (g L^−1^): ZnSO_4_·7H_2_O (0.001), MnSO_4_·7H_2_O (0.002), H_3_BO_3_ (0.01), Co(NO_3_)_2_·6H_2_O (0.001), Na_2_MoO_4_·2H_2_O (0.001), CuSO_4_·5H_2_O (0.0005·10^−3^), FeSO_4_·7H_2_O (0.7), and EDTA (Titriplex III, Merck, Darmstadt, Germany) (0.8). *A. platensis* was preserved in “Spirulina medium” [82] and *E. gracilis* in “Euglena medium with minerals”, which contained (g L^−1^): KNO_3_ (0.2), (NH_4_)_2_HPO_4_ (0.02), MgSO_4_·H_2_O (0.01), yeast extract (0.02), 30% *v*/*v* soil extract and 0.1% *v*/*v* of the same trace metals solution. Ultrapure water was used for medium preparation in all cases, except for *C. minutissima* and *T. tetrathele*, which were cultivated in 100% and 50% *v*/*v* filtered and sterile seawater (Whatman GF/F, Cytiva, Little Chalfont, UK; pore size 0.7 μm), respectively. Cultures were incubated in a controlled environment at 25 °C, except for *C. fritschii*, which was maintained at 35 °C due to its thermophilic nature [83], under continuous cool white, fluorescent illumination, with a photon flux density of 25 μmol m^−2^ s^−1^.

### 3.2. Anaerobic Digestion Effluent (Digestate)

Digestate was kindly provided from an anaerobic digestion facility in Aetolia-Acarnania, a regional unit of central Greece, which processes agricultural waste such as manure, corn silage, and olive mill wastewater. After collection, pretreatment was performed including centrifugation at 15,000 rpm for 20 min (Hermle Z 32 HK, Hermle Labortechnik GmbH, Wehingen, Germany). The pretreated ADE was stored at 4 °C until further use. The physicochemical characteristics of raw and treated ADE are presented in Table 5.

### 3.3. Experimental Setup

The experimental design was established as a comparative screening of four distinct cultivation conditions, and the observed responses reflect the combined effect of the conditions defining each strategy rather than the isolated effect of a single operating parameter. Autotrophic cultivation in synthetic medium was used as the reference condition. Acetate supplementation was subsequently evaluated to assess the effect of an additional organic carbon source under mixotrophic cultivation. Anaerobic digestion effluent was investigated as a waste-derived cultivation medium to evaluate simultaneous nutrient recovery and PHA production, whereas nitrogen-depleted cultivation was used to assess the effect of nitrogen limitation on PHA accumulation.

To initiate each experiment, pre-cultures were prepared in 500 mL conical flasks with a working volume of 400 mL, using the appropriate synthetic medium, described before, under continuous white light with a correlated color temperature of 6000 K. Each flask was aerated with filtered atmospheric air (PolyVent 0.22 μm PTFE, Whatman, Cytiva, Little Chalfont, UK) at a flow rate of 0.5 L min^−1^ to supply carbon dioxide. At the late exponential phase, the resulting biomass was harvested and concentrated by centrifugation at 4500 rpm for 5 min (Hermle Z 366, Hermle Labortechnik GmbH, Wehingen, Germany). This autotrophically produced biomass was then used as inoculum (200–300 mg L^−1^) for subsequent experiments, including autotrophic cultivation, mixotrophic growth with acetic acid, and mixotrophic growth with 10% *v*/*v* digestate, where growth performance was evaluated. For screening under nitrogen-depleted conditions, the entire biomass obtained from these pre-cultures was used to inoculate nitrogen-depleted synthetic medium, to ensure sufficient biomass concentration for PHA quantification over the 31-day cultivation period.

During autotrophic screening, each microorganism was grown in the synthetic medium with samples collected every 2–3 days to monitor biomass production and nutrient removal. For mixotrophic experiments, the medium was supplemented either with 2 g L^−1^ acetate or with 10% *v*/*v* liquid digestate, and samples were regularly taken to assess growth and substrate utilization. In cultures of *A. platensis* with digestate, NaHCO_3_ of 10 g L^−1^ was added to enhance growth under alkaline conditions [84]. Digestate-based experiments also included evaluation of wastewater treatment efficiency through COD, TP, and TN removal. Bioplastic production was determined at the end of each autotrophic and mixotrophic experiment.

For the investigation of PHA accumulation, a modified nitrogen-free synthetic medium was used, and both bioplastic and glycogen/starch accumulation were monitored at specific intervals over a 31-day cultivation period.

**Figure 8:**
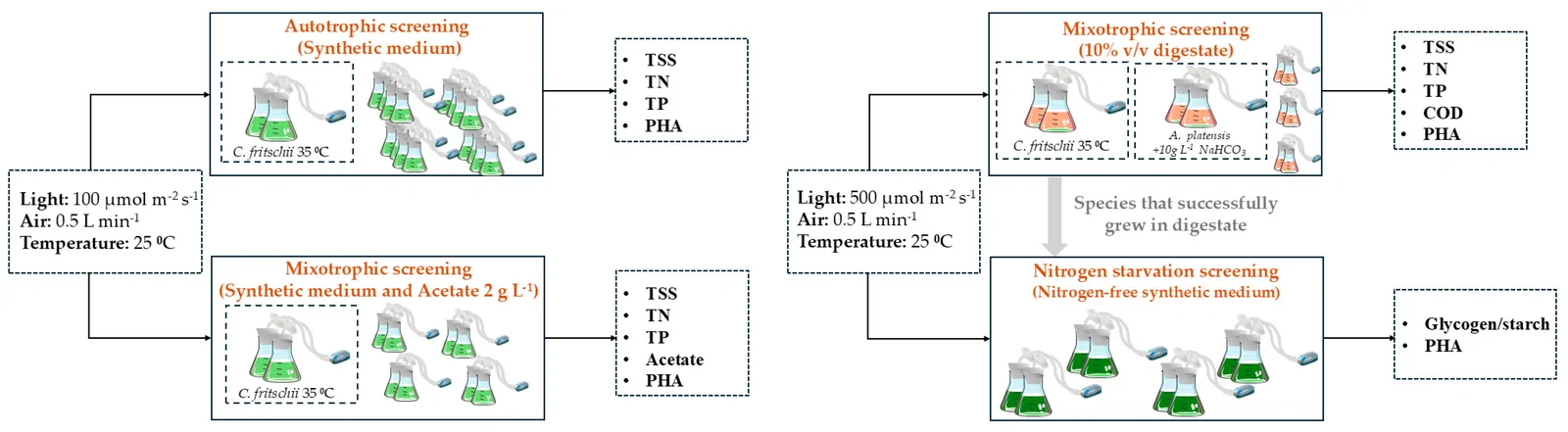
Experimental setup of the present study. Abbreviations: TSS, Total suspended solids; TN, Total nitrogen; TP, Total phosphorus; PHA, Polyhydroxyalkanoates.

All experiments were carried out in 500 mL flasks with a working volume of 400 mL at 25 ± 2 °C, except for *C. fritschii*, which was cultivated at 35 ± 2 °C. Aeration was maintained with filtered air at 0.5 L min^−1^. Continuous illumination was provided using white LED lights with a correlated color temperature of 6000 K, positioned beneath the flasks. For autotrophic and acetic acid mixotrophic conditions, light intensity was set at 100 μmol m^−2^ s^−1^, while for digestate and nitrogen-depleted cultures, it was increased to 500 μmol m^−2^ s^−1^ to account for medium characteristics and higher cell density, measured at the bottom surface of the flasks using a light meter (LI-250A, LI-COR, Lincoln, NE, USA). Figure 8 presents the experimental set up of the study.

### 3.4. Biomass Growth

To determine the biomass concentration (dry weight, DW) in the cultures throughout the experiment, the total suspended solids (TSS) method was applied [85]. TSS represents the mass of material retained on a filter after the removal of moisture. Filtration was carried out using GF/F filters with a pore size of 0.7 μm. The maximum specific growth rate (μ_max_, d^−1^) during the exponential growth phase was determined from the slope of the logarithmic plot of dry weight versus time.

### 3.5. Nutrients Measurements

Organic load, expressed as chemical oxygen demand (COD), and TP were determined according to Standard Methods [85]. TN was measured using the persulfate digestion method [85], followed by analysis with a Total Nitrogen Analyzer (TNM-1, Shimadzu, Kyoto, Japan). The consumption of acetate was monitored using gas chromatography (GC) with an Agilent Technologies 7890A system. Quantitative analysis was performed using a calibration curve prepared from standard solutions of acetic acid (Sigma-Aldrich), according to [86].

### 3.6. PHA Extraction and Quantification

The intracellular PHBV content was determined by quantifying its constituent monomer units, 3-hydroxybutyrate (3HB) and 3-hydroxyvalerate (3HV), using a gas chromatography-based analytical method after depolymerization and transesterification of the extracted PHA, as described by [87,88]. Briefly, 10–20 mg of dried biomass was subjected to acid-catalyzed methanolysis using chloroform and methanol containing 3% (*v*/*v*) H_2_SO_4_. Benzoic acid (2.5 g L^−1^) was used as an internal standard. Gas chromatography analysis was performed using a GC-FID system (Agilent Technologies 7890A, Santa Clara, CA, USA). Separation was achieved using a capillary column (DB-FFAP, 30 m length, 0.25 mm internal diameter, and 0.25 μm film thickness). The PHBV copolymer (403105, Sigma-Aldrich, Taufkirchen, Germany), with a 3HV content of 8 mol %, was used as a calibration standard. The limits of detection for 3HB and 3HV were estimated at 32.3 and 2.1 mg L^−1^, respectively, based on the calibration curves as 3.3σ/S, where σ represents the residual standard deviation of the regression and S is the slope of the calibration curve.

### 3.7. Glycogen/Starch Quantification

The main reserve polysaccharide in eukaryotic microalgae is starch, whereas cyanobacteria predominantly accumulate glycogen [62]. Glycogen/starch content was determined according to the method described by [89], with slight modifications. Specifically, carbohydrate concentration, expressed as glucose equivalents, was measured photometrically following an analytical procedure involving l-tryptophan, sulfuric acid, and boric acid [90]. Although glycogen/starch is synthesized intracellularly, exopolysaccharides (EPS) may also be produced and detected by this method. Therefore, both unfiltered (raw) samples and filtered samples (0.22 μm nylon syringe filter, Labfil, ALWSCI, Hangzhou, China) were analyzed. Glycogen/starch concentration was determined according to Equation (1).

Glycogen/starch [mg L^−1^] = Carbohydrates_raw sample_ [mg L^−1^] – Carbohydrates_filtrate sample_ [mg L^−1^] (1)

### 3.8. Statistical Analysis

All experiments were performed in duplicate, and the results are presented as average values along with the standard deviation. One-way analysis of variance (ANOVA) and Tukey’s test were performed to examine the statistical difference between nutrients removal and bioplastic accumulation for each screening experiment. Significance was established at *p* < 0.05 (null hypothesis). A two-way ANOVA was applied to evaluate the effect of species and cultivation conditions, as well as their interaction, on the examined parameters (μ_max_, maximum biomass concentration, and PHBV content). Experimental conditions where species failed to grow or where measurements were below the detection limit were treated as zero values. The significance of main and interaction effects was determined at a 95% confidence level (*p* < 0.05). The analysis was performed via Minitab 18 software.

## 4. Conclusions

To build a more sustainable future, it is essential to shift toward the use of biodegradable bioplastics produced from renewable resources. The comprehensive screening applied in this study provides valuable insights by investigating a wide range of microalgae and cyanobacteria under different cultivation conditions. Acetate supplementation strongly enhanced PHBV accumulation in selected cyanobacteria, particularly *Synechocystis* sp. and *A. platensis*. Most of the photosynthetic microorganisms managed to adapt and grow in anaerobic digestion effluent, enabling its bioremediation with substantial removal of nitrogen, phosphorus, and organic matter. However, bioplastic accumulation remained relatively low. In contrast, nitrogen-depleted conditions provided the strongest induction of PHBV accumulation, with *Synechocystis* sp. reaching the highest biopolymer content observed in the study. These findings suggest that an integrated two-stage cultivation strategy could be particularly promising. Cultivating photosynthetic microorganisms in anaerobic digestion effluents could support biomass production and wastewater bioremediation. Inoculating the produced biomass to nitrogen starvation could trigger intracellular carbon storage and enhance PHA accumulation. This approach could combine wastewater treatment and nutrient recovery with bioplastic production, improving the overall sustainability of the process. Although cultivation conditions significantly affected PHBV accumulation, their impact was strongly dependent on the photosynthetic microorganism, with cyanobacteria generally showing greater potential for bioplastic accumulation than microalgae. Identifying the most promising photosynthetic microorganisms and cultivation conditions is the first step in bioplastic production from microalgae and cyanobacteria. Further research into both the upstream and downstream processes is essential to ensure the environmental sustainability and economic feasibility of the process.

## Figures and Tables

**Table 1:** Nutrients removal (%) during autotrophic cultivation.

Species	TN_removal_ (%)	TP_removal_ (%)
*C. vulgaris*	28.5 ± 3.6 ^d,e^	98.6 ± 0.1 ^a^
*E. gracilis*	65.2 ± 6.3 ^a^	86.5 ± 5.5 ^b,c^
*C. oleofaciens*	37.8 ± 3.3 ^b,c,d,e^	97.9 ± 0.0 ^a,b^
*A. obliquus*	44.2 ± 6.6 ^a,b,c,d,e^	94.0 ± 1.7 ^a,b^
*P. kessleri*	45.2 ± 3.3 ^a,b,c,d,e^	98.6 ± 0.2 ^a^
*B. braunii*	30.9 ± 2.5 ^c,d,e^	91.6 ± 2.7 ^a,b^
*C. vacuolata*	26.7 ± 3.8 ^e^	98.5 ± 0.1 ^a^
*N. muscorum*	55.7 ± 5.8 ^a,b^	90.6 ± 5.2 ^a,b^
*Synechocystis* sp.	52.5 ± 6.8 ^a,b,c^	90.4 ± 0.8 ^a,b^
*C. minutissima*	35.6 ± 11.1 ^b,c,d,e^	96.8 ± 2.2 ^a,b^
*C. fritschii*	46.0 ± 1.9 ^a,b,c,d,e^	78.3 ± 3.4 ^c^
*C. zofigiensis*	36.0 ± 6.5 ^b,c,d,e^	96.8 ± 0.4 ^a,b^
*T. tetrathele*	41.7 ± 4.5 ^b,c,d,e^	94.7 ± 0.5 ^a,b^
*A. platensis*	49.4 ± 2.3 ^a,b,c,d^	55.1 ± 5.6 ^d^

^a,b,c,d,e^ Data in the same column that do not share a letter are significantly different based on Tukey test (*p* < 0.05).

**Table 2:** Nutrients removal (%) during mixotrophic cultivation with acetate.

Species	Acetate_removal_ (%)	TN_removal_ (%)	TP_removal_ (%)
*C. vulgaris*	32.8 ± 4.4 ^e,f^	73.6 ± 3.5 ^a,b^	70.0 ± 5.6 ^a,b,c^
*E. gracilis*	98.4 ± 0.2 ^a^	79.4 ± 7.3 ^a^	76.4 ± 17.6 ^a,b,c^
*C. oleofaciens*	18.2 ± 8.7 ^f,g^	70.8 ± 5.5 ^a,b^	74.4 ± 7.2 ^a,b,c^
*A. obliquus*	100.0 ± 0.0 ^a^	58.7 ± 5.0 ^b,c^	63.6 ± 10.4 ^b,c^
*P. kessleri*	9.1 ± 2.5 ^g^	58.2 ± 4.1 ^b,c^	82.8 ± 10.0 ^a,b,c^
*B. braunii*	98.4 ± 0.4 ^a^	57.8 ± 6.5 ^b,c^	77.2 ± 6.0 ^a,b,c^
*C. vacuolata*	59.6 ± 5.0 ^c,d^	41.5 ± 0.7 ^c^	85.8 ± 1.3 ^a,b^
*N. muscorum*	100.0 ± 0.0 ^a^	43.5 ± 6.4 ^c^	80.9 ± 8.8 ^a,b,c^
*Synechocystis* sp.	100.0 ± 0.0 ^a^	50.0 ± 1.4 ^c^	84.4 ± 4.5 ^a,b^
*C. minutissima*	98.5 ± 0.1 ^a^	74.0 ± 2.2 ^a,b^	95.6 ± 2.8 ^a^
*C. fritschii*	75.0 ± 0.3 ^b,c^	82.7 ± 2.6 ^a^	85.0 ± 1.4 ^a,b^
*C. zofigiensis*	60.2 ± 2.8 ^c,d^	39.1 ± 9.6 ^c^	84.4 ± 9.2 ^a,b,c^
*T. tetrathele*	51.7 ± 16.5 ^d,e^	87.2 ± 3.8 ^a^	94.8 ± 4.8 ^a,b^
*A. platensis*	96.3 ± 0.6 ^a,b^	58.5 ± 2.1 ^b,c^	52.8 ± 4.1 ^c^

^a,b,c,d,e,f,g^ Data in the same column that do not share a letter are significantly different based on Tukey test (*p* < 0.05).

**Table 3:** Nutrients removal (%) during mixotrophic cultivation in 10% *v*/*v* anaerobic digestion effluent.

Species	COD_removal_ (%)	TN_removal_ (%)	TP_removal_ (%)
*C. vulgaris*	50.9 ± 8.1 ^a,b^	93.6 ± 2.6 ^a^	92.5 ± 1.1 ^a,b^
*E. gracilis*	35.3 ± 2.5 ^a,b,c^	58.2 ± 4.2 ^c^	90.5 ± 1.2 ^a,b^
*C. oleofaciens*	35.3 ± 8.4 ^a,b,c^	91.9 ± 2.0 ^a^	100.0 ± 0.0 ^a^
*A. obliquus*	53.4 ± 6.9 ^a^	93.9 ± 2.9 ^a^	100.0 ± 0.0 ^a^
*P. kessleri*	21.1 ± 8.2 ^b,c^	92.0 ± 2.4 ^a^	100.0 ± 0.0 ^a^
*B. braunii*	18.7 ± 7.9 ^c^	81.3 ± 1.6 ^b^	91.6 ± 0.9 ^a,b^
*C. vacuolata*	50.0 ± 11.7 ^a,b^	98.2 ± 0.4 ^a^	67.7 ± 3.1 ^c^
*N. muscorum*	61.6 ± 9.3 ^a^	94.2 ± 4.3 ^a^	56.4 ± 3.1 ^c^
*Synechocystis* sp.	59.6 ± 1.1 ^a^	94.7 ± 0.5 ^a^	83.0 ± 10.4 ^b^
*C. minutissima*	36.8 ± 7.8 ^a,b,c^	97.9 ± 0.9 ^a^	100.0 ± 0.0 ^a^
*C. fritschii*	21.0 ± 4.8 ^b,c^	99.8 ± 0.6 ^a^	50.0 ± 4.6 ^c^

^a,b,c^ Data in the same column that do not share a letter are significantly different based on Tukey test (*p* < 0.05).

**Table 4:** Maximum growth rate (µ_max_, d^−1^), maximum biomass (g L^−1^), and PHBV content (% *w*/*w*) of microalgal and cyanobacterial species during the different screening experiments. Color gradients indicate the relative magnitude of each parameter, with lighter shades indicating lower values and darker shades indicating higher values.

Species	μ_max_ (d^−1^)			Max. Biomass (g L^−1^)			PHBV (% *w*/*w*)			
	Autotrophic	Acetate	Digestate	Autotrophic	Acetate	Digestate	Autotrophic	Acetate	Digestate	N-Starvation
*C. vulgaris*	0.40 ± 0.00	0.72 ± 0.04	0.48 ± 0.13	1.39 ± 0.04	0.69 ± 0.04	1.09 ± 0.04	1.1 ± 0.1	0.8 ± 0.1	n.d	0.2 ± 0.0
*E. gracilis*	0.44 ± 0.01	0.82 ± 0.08	0.28 ± 0.01	0.75 ± 0.04	0.43 ± 0.06	0.70 ± 0.09	0.3 ± 0.0	1.2 ± 0.1	n.d	0.9 ± 0.1
*C. oleofaciens*	0.53 ± 0.02	0.46 ± 0.03	0.12 ± 0.02	1.79 ± 0.07	0.74 ± 0.02	1.13 ± 0.10	0.5 ± 0.0	n.d **	n.d	0.7 ± 0.0
*C. minutissima*	0.33 ± 0.06	0.51 ± 0.01	0.19 ± 0.03	3.38 ± 0.80	1.85 ± 0.57	2.60 ± 0.17	0.6 ± 0.3	n.d	n.d	0.8 ± 0.1
*C. zofigiensis*	0.45 ± 0.02	1.21 ± 0.37	- *	1.08 ± 0.07	0.76 ± 0.21	-	0.3 ± 0.0	n.d	n.d	n.a ***
*A. obliquus*	0.37 ± 0.00	0.37 ± 0.01	0.34 ± 0.02	1.78 ± 0.18	1.10 ± 0.21	0.94 ± 0.18	0.6 ± 0.1	n. d	n.d	0.7 ± 0.1
*T. tetrathele*	0.45 ± 0.01	0.43 ± 0.02	-	1.16 ± 0.20	1.88 ± 0.50	-	0.3 ± 0.1	n.d	n.d	n.a
*P. kessleri*	0.45 ± 0.01	0.82 ± 0.11	0.41 ± 0.02	2.32 ± 0.03	1.05 ± 0.05	0.75 ± 0.05	0.9 ± 0.1	n.d	n.d	0.73 ± 0.06
*B. braunii*	0.40 ± 0.02	0.71 ± 0.08	0.14 ± 0.03	0.92 ± 0.06	0.50 ± 0.01	0.95 ± 0.10	1.2 ± 0.4	2.4 ± 0.4	0.60 ± 0.14	1.4 ± 0.1
*A. platensis*	0.49 ± 0.04	0.62 ± 0.16	-	2.20 ± 0.14	1.91 ± 0.19	-	4.3 ± 0.3	9.9 ± 0.4	n.d	n.a
*C. vacuolata*	0.27 ± 0.01	0.39 ± 0.04	0.15 ± 0.01	1.82 ± 0.17	1.51 ± 0.19	1.19 ± 0.09	0.8 ± 0.1	n.d	n.d	1.0 ± 0.1
*N. muscorum*	0.48 ± 0.01	0.58 ± 0.08	0.12 ± 0.03	0.68 ± 0.11	0.49 ± 0.01	0.53 ± 0.07	0.2 ± 0.0	2.4 ± 0.9	0.8 ± 0.1	0.6 ± 0.1
*C. fritschii*	0.52 ± 0.02	0.70 ± 0.02	0.09 ± 0.01	1.33 ± 0.30	0.92 ± 0.19	1.20 ± 0.14	1.8 ± 0.0	4.9 ± 0.5	1.3 ± 0.1	4.1 ± 0.6
*Synechocystis* sp.	1.20 ± 0.14	0.40 ± 0.02	0.19 ± 0.02	1.10 ± 0.05	1.00 ± 0.11	0.50 ± 0.01	1.1 ± 0.1	10.2 ± 1.5	1.0 ± 0.2	14.4 ± 1.3

* -: species did not grow under the applied conditions, ** n.d: not detected, *** n.a: not applied in this condition.

**Table 5:** Physicochemical characteristics of raw and treated digestate. Data are presented as means ± SD (*n* = 3).

Parameters	Raw	Treated
pH	8.46 ± 0.02	8.65 ± 0.05
OD_550_ (Au cm^−1^)	28.93 ± 0.53	2.15 ± 0.04
Total suspended solids (TSS) (g L^−1^)	12.35 ± 0.49	0.15 ± 0.03
Chemical oxygen demand (COD) (g L^−1^)	11.08 ± 1.50	4.18 ± 0.14
Total nitrogen (TN) (g L^−1^)	6.70 ± 0.04	3.78 ± 0.07
Total phosphorus (TP) (g L^−1^)	0.29 ± 0.10	0.03 ± 0.00

## Data Availability

Data will be made available upon request.

## Abbreviations

The following abbreviations are used in this manuscript:

PHA	Polyhydroxyalkanoates
PHB	Poly(3-hydroxybutyrate)
PHBV	Poly(3-hydroxybutyrate-*co*-3-hydroxyvalerate)
TSS	Total suspended solids
COD	Chemical oxygen demand
TN	Total nitrogen
TP	Total phosphorus
ATP	Adenosine triphosphate
NADPH	Nicotinamide adenine dinucleotide phosphate

## Appendix A

**Table A1:** Two-way ANOVA evaluating the effects of species, cultivation condition, and their interaction on maximum specific growth rate (μ_max_), maximum biomass concentration, and PHBV content. The analysis included all 14 species tested under the three cultivation conditions common to all species: autotrophic cultivation, mixotrophic cultivation with acetate, and cultivation in anaerobic digestion effluent. Percentage contributions were calculated from the adjusted sums of squares relative to the total sum of squares.

Source of Variation	μ_max_		Max. Biomass		PHBV	
	Contribution (%)	*p*-Value	Contribution (%)	*p*-Value	Contribution (%)	*p*-Value
Species	13.29	<0.001	49.45	<0.001	43.28	<0.001
Cultivation condition	45.42	<0.001	17.97	<0.001	13.43	<0.001
Species × Cultivation condition	37.11	<0.001	28.24	<0.001	42.33	<0.001

**Table A2:** Two-way ANOVA evaluating the effects of species, cultivation conditions, and their interaction on PHBV content when nitrogen-starvation was included as an additional cultivation condition. The analysis was restricted to the 11 species tested under all four conditions. Percentage contributions were calculated from the adjusted sums of squares relative to the total sum of squares.

Source of Variation	PHBV	
	Contribution (%)	*p*-Value
Species	48.79	<0.001
Cultivation condition	9.55	<0.001
Species × Cultivation condition	40.67	<0.001
